# A pore way to heal and regenerate: 21st century thinking on biocompatibility

**DOI:** 10.1093/rb/rbw006

**Published:** 2016-02-18

**Authors:** Buddy D. Ratner

**Affiliations:** Department of Bioengineering and Chemical Engineering, University of Washington Engineered Biomaterials, University of Washington, Seattle, WA 98195, USA

**Keywords:** biocompatibility, foreign body reaction, pore, healing, regeneration

## Abstract

This article raises central questions about the definition of biocompatibility, and also about how we assess biocompatibility. We start with the observation that a porous polymer where every pore is spherical, ∼40 microns in diameter and interconnected, can heal into vascularized tissues with little or no fibrosis and good restoration of vascularity (i.e., little or no foreign body reaction). The same polymer in solid form will trigger the classic foreign body reaction characterized by a dense, collagenous foreign body capsule and low vascularity. A widely used definition of biocompatibility is ‘the ability of a material to perform with an appropriate host response in a specific application’. With precision-porous polymers, in direct comparison with the same polymer in solid form, we have the same material, in the same application, with two entirely different biological reactions. Can both reactions be ‘biocompatible?’ This conundrum will be elaborated upon and proposals will be made for future considerations and measurement of biocompatibility.

## Introduction

Medical implants made of synthetic materials or modified natural materials are used in millions of procedures each year worldwide, save millions of lives and improve the quality of life for millions more (Table 1). A central concern with all these devices is biocompatibility. But the definitions we have of biocompatibility are fraught with ambiguity and imprecision hampering our ability to use this term to make accurate statements about the healing and integration of medical devices. This article will examine biocompatibility in the early 21st century and offer suggestions for a definition of biocompatibility that will be more useful and accurate.

**Table 1.** . Estimates of number of medical devices used worldwide each year

Intraocular lenses	∼14 million
Contact lenses	125 million
Vascular grafts	∼400 000
Hip and knee prostheses	2 million
Catheters	>1 billion
Heart valves	300 000
Stents (cardiovascular)	>1 million
Breast implants	∼600 000
Dental implants	3 million
Pacemakers	600 000
Renal dialyzers	∼1.2 million
Left ventricular assist devices	>20 000

A definition of biocompatibility as applied to medical devices is ‘the ability of a material to perform with an appropriate host response in a specific application’ [1].

This definition, though accurate, offers little insight into the meaning of the word—e.g., it offers no guidance into how to test for biocompatibility or how to improve the biocompatibility of a material or device.

**Figure 1.**
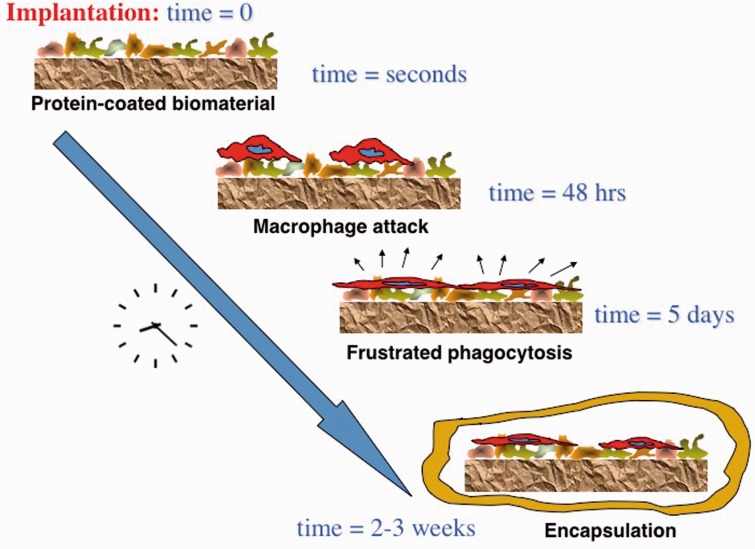
. Time course of the FBR to implanted materials.

Let us start with a consideration of the bioreaction to an implanted ‘biocompatible’ biomaterial or medical device. The outcome observed (after about 2 weeks of implantation in a mammal), the foreign body reaction (FBR), has been reported since well before 1970 [2] and is characterized by the implant surrounded by a dense, collagenous capsule, low vascularity in and around the capsule and inflammatory cells at the implant surface. Figure 1 suggests the etiology of the FBR seen in most of the biocompatible implants.

Because this FBR is ubiquitous for implanted materials, and because we put millions of devices into humans every year with all devices that are implanted for over 2 weeks exhibiting this reaction, why should there be a concern? In spite of our overall success with medical implants, there are hundreds of thousands of reports each year to the regulatory agencies of complications or adverse outcomes with implanted medical devices (in the year 2015, the United States Food and Drug Administration (US FDA) received well over 900 000 adverse event reports). Furthermore, the FBR capsule is associated with walling off of implanted electrodes (impeding electrical signals), walling off of implanted biosensors, walling off of drug delivery systems, capsular problems around breast implants, fibrotic occlusion of glaucoma drains, poor healing of devices in bone, highly encapsulated pacemaker lead healing that complicates revision surgeries, vascular graft sheaths that may inhibit endothelialization, device centered infection and many other complications as well. How many of those 900 000 reports of adverse events might be attributed to poor (fibrotic) healing increasing the chance of infection, device failure, device extrusion and other complications?

Given the concerns associated with the FBR, why do we accept this FBR as biocompatible? We base our acceptance of the FBR on ISO standards deeming this reaction as acceptable and regulatory agency approvals that equate the uncomplicated FBR with nontoxic and inert. If the implant, after ∼30 days, is found in a thin (50–200 micron) fibrous capsule with little or no additional evidence of reaction at the implant site, we call it biocompatible. Yet, instead of the body accepting the implant (biocompatible) the body is isolating the implant as an object to be removed from contact with active systems within the body.

## Implants that heal without the foreign body reaction

As early as 1973 it was demonstrated that the same material in a solid form or in a porous form will heal differently after implantation with more blood vessels around the porous material [3]. Although many papers on this subject were published in the years following that 1973 study, the relationships between pore size, pore structure and healing were never clearly established.

**Figure 2.**
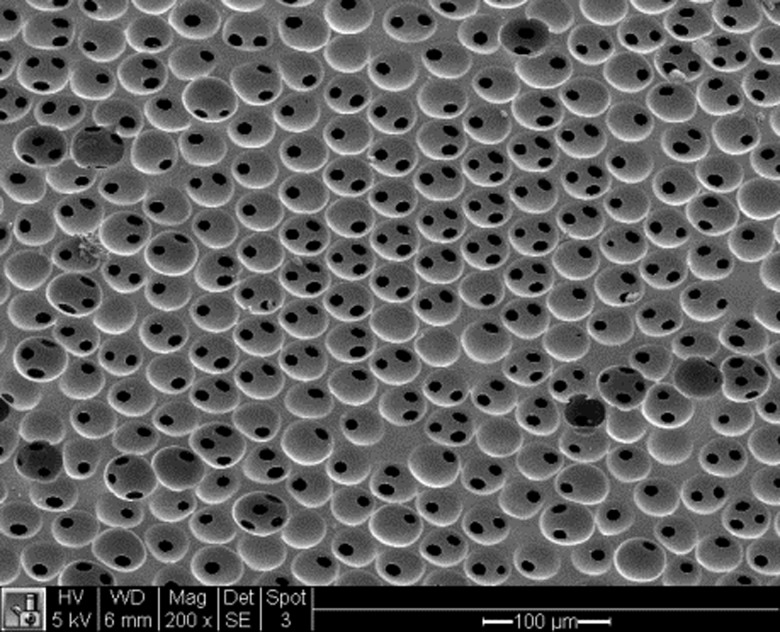
. A scanning electron microscopy cross-section through a precision-porous biomaterial where every pore is the same size and pores are interconnected (image by Kelsey Willson).

In the early 1990s, the University of Washington Engineered Biomaterials program at the University of Washington set out to better define this relationship. A class of materials was developed where every pore was spherical, the same size and pores were interconnected (Fig. 2).

Upon implantation, it was noted that materials with pores in 30–40 micron range healed with vigorous new blood vessel growth and little fibrosis [4, 5]. Materials with larger and smaller pores had fewer blood vessels and more fibrosis. Such porous materials with 30–40 micron pores were found to heal well (i.e., nonfibrotic, vascularized, reconstructive) into skin [6], heart [7], bone, sclera and vaginal wall. A common feature of these proregenerative materials is that they stimulate invading macrophages to the M2 (proregenerative) polarization [5, 7–9]. Other strategies have also demonstrated nonfibrotic healing and associated M2 macrophages [10, 11].

## Implications of materials that heal without the foreign body reaction

Comparing the healing of a solid slab of cross-linked poly(2-hydroxyethyl methacrylate) to the same polymer with 40 micron pores, we see two different bioreactions: fibrotic, avascular isolation of the solid implant versus nonfibrotic, vascularized tissue integration of the porous implant [5]. Yet, the only word we have for these two tissue reactions is biocompatible. The healing reactions are so different—how can we call both biocompatible?

This inconsistency in our ability to accurately describe these healing reactions leads to the proposal of two definitions for the biological reaction to implanted materials:

Biocompatibility: The ability of materials to locally trigger and guide normal wound healing, reconstruction and tissue integration.

Biotolerability: The ability of materials to reside in the body for long periods of time with only low degrees of inflammatory reaction.

The millions of medical devices that now are routinely used in clinical medicine and have regulatory agency approval are tolerated by the body. This is ‘biotolerability’. A future generation of devices will be made from materials that can seamlessly integrate into tissues in a vascularized, nonfibrotic manner and will be genuinely compatible (integrative) with living organisms—this is biocompatibility.

**Figure 3.**
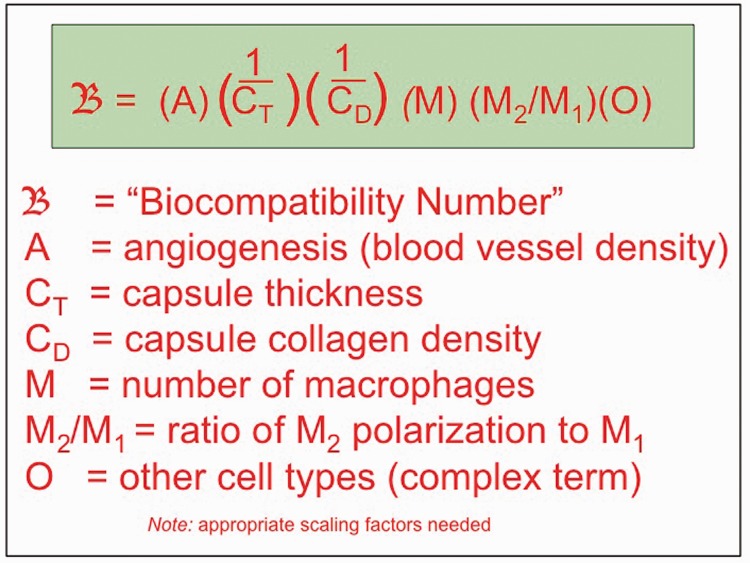
. A suggestion for an equation to quantitatively express biocompatibility.

To further refine the definition of biocompatibility, we can envision a quantitative expression that will allow us to understand just how biocompatible a biomaterial is (are all biomaterials equally biocompatible?), and provide a basis for improving or optimizing biocompatibility. Such an equation is suggested in Fig. 3.

The use of an equation such as suggested in Fig. 3 necessitates new assays and immunological stains needed to acquire the quantitative data from the explanted implant. Some of the parameters such as angiogenesis might be further qualified by considering the size of new vessels formed and the quality of those blood vessels. Furthermore, each of the terms in the equation may not contribute equally to the phenomenon called biocompatibility, so scaling factors may be necessary.

## Conclusions

The field of biomaterials has made possible medical devices that save millions of lives and improve the quality of life for millions more. However, there are large numbers of adverse outcomes in the healing of such implants and many procedures are impeded by fibrotic, nonintegrative healing. The potential now exists to bypass the FBR and achieve a reconstructive, integrative healing that may minimize complications. However, the assessment methods we now use for the routine measurement of biocompatibility of biomaterials and devices are inadequate for measuring these new, desirable outcomes. Similarly, our vocabulary for expressing these nonfibrotic, proregenerative outcomes falls short of giving us accurate terminology to describe these outcomes. This article proposes new definitions that describe a ‘biocompatibility of the future’ and still permits us to discuss the fibrotic healing of today’s medical devices. In addition, thoughts are presented on a quantitative expression for biocompatibility that may permit us to rank the performance of implant materials and guide the development of improved biomaterials.

## References

[1] Williams DF. Definitions in Biomaterials: Progress in Biomedical Engineering, Vol. 4 Amsterdam: Elsevier, 1987.

[2] Homsy CA. Bio-compatibility in selection of materials for implantation. J Biomed Mater Res 1970;4:341–56. 5469182 10.1002/jbm.820040306

[3] Karp RD Johnson KH Buoen LC Tumorigenesis by Millipore filters in mice: histology and ultrastructure of tissue reactions as related to pore size. J Natl Cancer Ins 1973;51:1275–9. 10.1093/jnci/51.4.1275 4583375

[4] Marshall AJ Irvin CA Barker T Biomaterials with tightly controlled pore size that promote vascular in-growth. ACS Polym Prepr 2004;45:100–1.

[5] Sussman EM Halpin MC Muster J Porous implants modulate healing and induce shifts in local macrophage polarization in the foreign body reaction. Ann Biomed Eng 2014;42:1508–16. 24248559 10.1007/s10439-013-0933-0

[6] Fukano Y Usui ML Underwood RA Epidermal and dermal integration into sphere-templated porous poly(2-hydroxyethyl methacrylate) implants in mice. J Biomed Mater Res A 2010;94:1172–86. 20694984 10.1002/jbm.a.32798 PMC2998188

[7] Madden LR Mortisen DJ Sussman EM Proangiogenic scaffolds as functional templates for cardiac tissue engineering. Proc Natl Acad Sci U S A 2010;107:15211–6. 20696917 10.1073/pnas.1006442107 PMC2930533

[8] Brown BN Ratner BD Goodman SB Macrophage polarization: an opportunity for improved outcomes in biomaterials and regenerative medicine. Biomaterials 2012;33: 3792–802. 22386919 10.1016/j.biomaterials.2012.02.034 PMC3727238

[9] Mantovani A. Macrophage diversity and polarization: in vivo veritas. Blood 2006;108:408–9.

[10] Zhang L Cao Z Bai T Zwitterionic hydrogels implanted in mice resist the foreign-body reaction. Nat Biotechnol 2013;31:553–6. 23666011 10.1038/nbt.2580

[11] Badylak SF. Decellularized allogeneic and xenogeneic tissue as a bioscaffold for regenerative medicine: factors that influence the host response. Ann Biomed Eng 2014;42:1517–27. 24402648 10.1007/s10439-013-0963-7

